# A rare case of Meckel’s diverticulum causing small bowel obstruction in a 50-year-old man

**DOI:** 10.1016/j.ijscr.2020.01.047

**Published:** 2020-02-06

**Authors:** Lei Ying, Jeong-moh John Yahng

**Affiliations:** Department of General Surgery, Western Health, Victoria, Australia

**Keywords:** Meckel’s diverticulum, Mesodiverticular adhesion, Small bowel obstruction

## Abstract

**Introduction:**

Meckel’s diverticulum is the most common congenital anomaly of the small intestine. Common complications involving Meckel’s diverticulum include hemorrhage, intestinal obstruction, and inflammation.

**Presentation of case:**

We present a rare case of a Meckel’s diverticulum causing small bowel obstruction. A 50-year old male presented to the emergency department (ED) with vomiting, abdominal pain and distension. Computed tomography (CT) of the abdomen showed dilated small bowel loops consistent with a small bowel obstruction. The patient was taken to the operating theatre for a laparotomy and was found to have a transition point from a mesodiverticular adhesion causing upstream dilatation which was released. The patient recovered with no postoperative complications and was discharged home.

**Discussion:**

Meckel’s diverticulum is the most common congenital anomaly of the small intestine. Diagnosis of Meckel’s diverticulum is difficult to confirm preoperatively as most patients are asymptomatic. Frequent complications of Meckel’s diverticulum include hemorrhage, intestinal obstruction, and infection, with intestinal obstruction being the second most common complication.

**Conclusion:**

The complications of Meckel’s diverticulum should be considered by the treating clinician in the differential diagnosis of small bowel obstruction.

## Highlights

• Meckel’s diverticulum is the most prevalent congenital abnormality of the gastrointestinal tract.
• Diagnosis of a Meckel’s diverticulum is difficult and often incidental due to the absence of symptoms in most patients.
• The complications of Meckel’s diverticulum should be considered by the treating clinician in the differential diagnosis of small bowel obstruction.

## 1 Introduction

Small bowel obstruction accounts for 20% of all acute surgical admissions with the most common cause being postoperative adhesions followed by hernias. Meckel's diverticulum is the most common congenital anomaly of the gastrointestinal tract. It results from incomplete obliteration of the vitelline duct leading to the formation of a true diverticulum of the small intestine which is usually located on the antimesenteric border of the ileum [1]. Meckel's diverticulum occurs in approximately 1%–3% of the population with a male-to-female ratio of 2:1, is located approximately 60 cm, but up to 100 cm from the ileocecal valve, and can be between 1–10 cm in length [2].

Approximately 3% of patients develop a complication over the course of their lives, typically before the age of 2, but up to 80 years of age. Symptomatic Meckel's diverticula most often contain both native intestinal and heterotropic gastric mucosa. There is no familial predisposition for Meckel’s diverticulum, but the prevalence is increased in children with major malformations of the umbilicus, alimentary tract, nervous system, or cardiovascular system [3].

Most of the Meckel’s diverticula are discovered incidentally during a surgical procedure performed for other reasons. Hemorrhage, small bowel obstruction, and infection (diverticulitis) are the most frequent complications [4].

Histologically, heterotopic gastric and pancreatic mucosas are frequently observed in the diverticula of symptomatic patients. Involvement of the mesentery or mesodiverticular band of the diverticulum is rarely seen. We present the following case in compliance with the SCARE criteria of surgical case report guidelines [5].

## 2 Case report

A 50-year old man of Indian descent with no prior medical or surgical history presented to the Emergency Department (ED) with 3 days of vomiting and lower abdominal pain. He had a raised white cell count (WCC) of 14.5 × 10^9^/L and a C-reactive protein (CRP) of 14. Because he passed some loose stools with his vomiting, he was presumed to have gastroenteritis by the ED treating team and observed in the short stay unit (SSU). On the next day, he developed abdominal distension, his vomiting and abdominal pain was worse, so was referred to the surgical team for review.

**Fig. 1:**
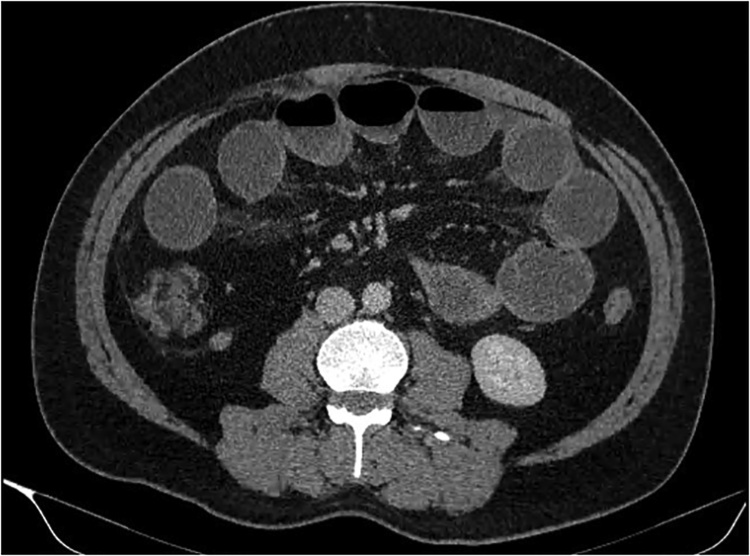
Axial view of the CT abdomen and pelvis showing multiple dilated small bowel loops consistent with SBO.

**Fig. 2:**
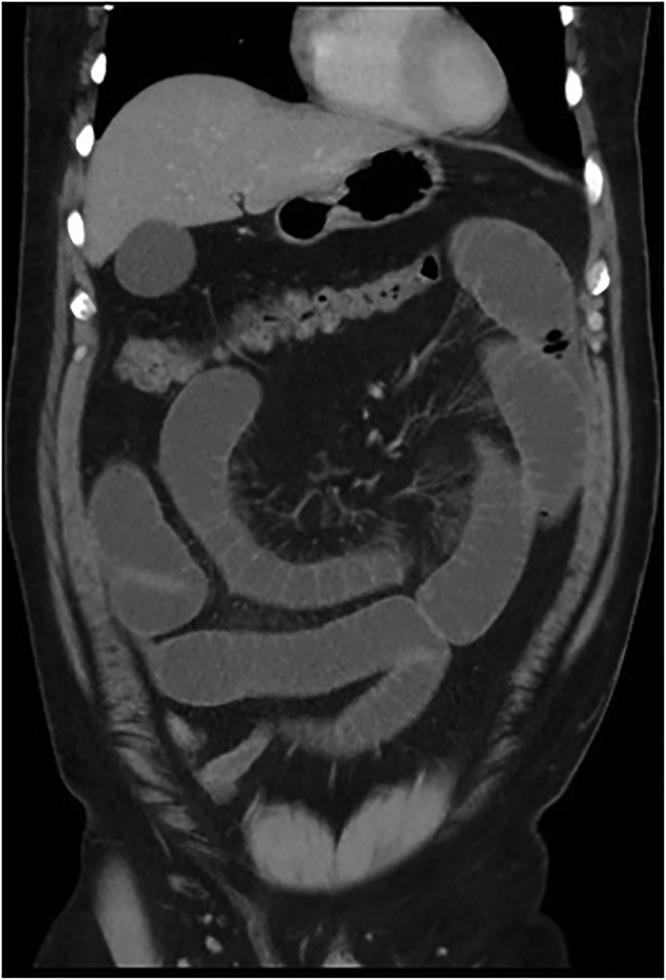
Coronal view of the CT abdomen and pelvis showing multiple dilated small bowel loops consistent with SBO.

**Fig. 3:**
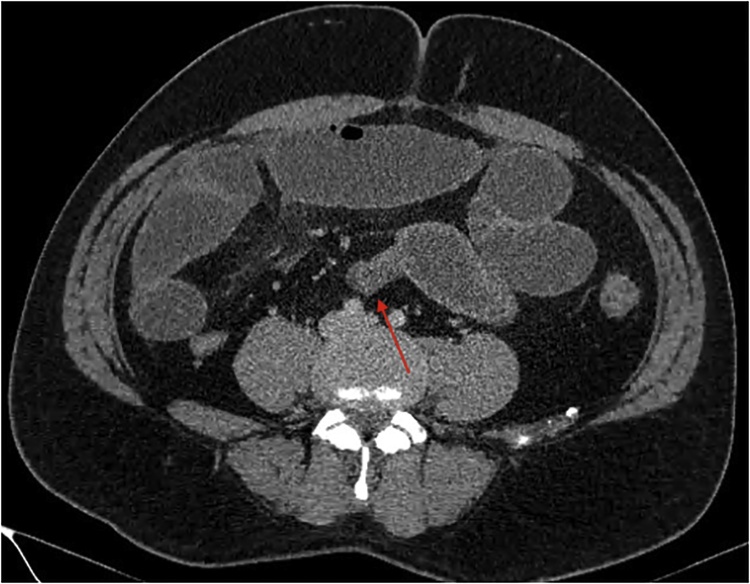
Axial view of the CT abdomen and pelvis showing transition point of the SBO with the angulation of the small bowel suggesting a kink.

**Fig. 4:**
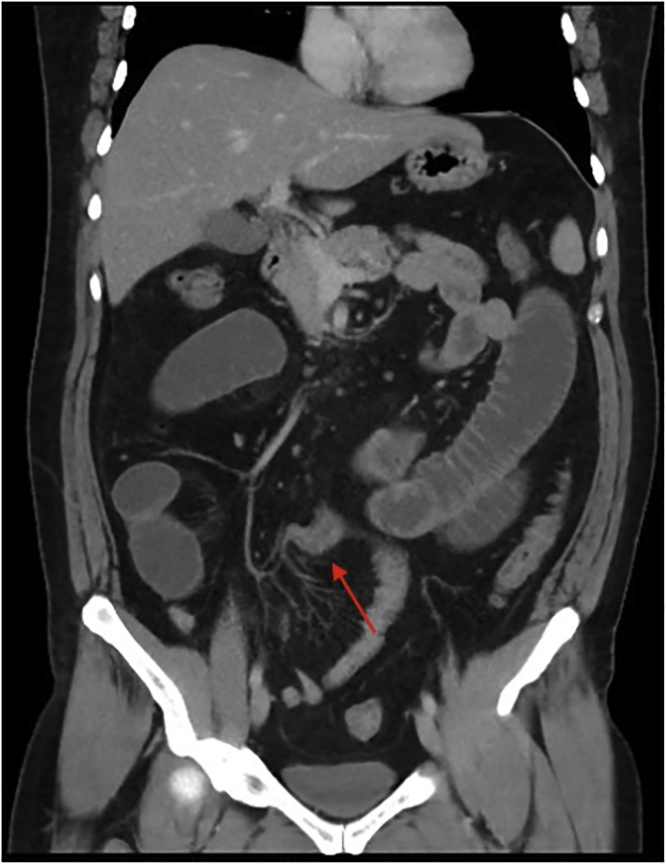
Coronal view of the CT abdomen and pelvis showing transition point of the SBO with the angulation of the small bowel suggesting a kink.

**Fig. 5:**
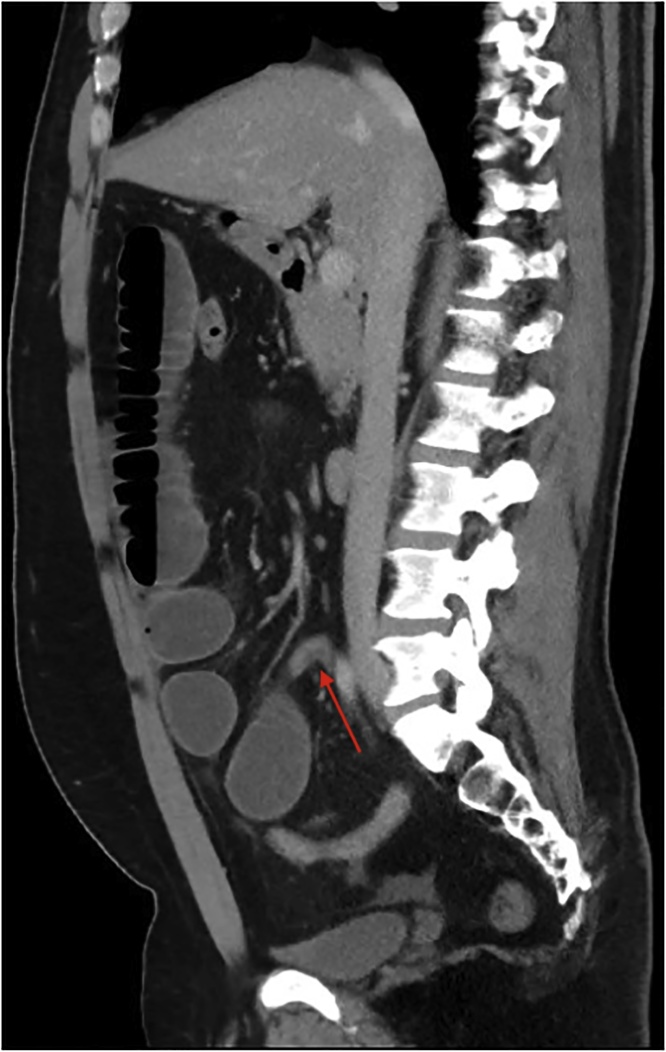
Sagittal view of the CT abdomen and pelvis showing transition point of the SBO with the angulation of the small bowel suggesting a kink.

A plain film chest and abdominal X-ray showed a dilated stomach and multiple air-fluid levels respectively. Computed tomography (CT) of the abdomen and pelvis demonstrated multiple markedly distended and fluid-filled small bowel loops throughout the abdomen (Figs. 1, 2) with a transition point within the right lower quadrant suggestive of adhesions (Fig. 3, Fig. 4, Fig. 5). He was urgently taken to the operating theatre on the diagnosis of adhesive small bowel obstruction (SBO) in a “virgin abdomen”.

**Fig. 6:**
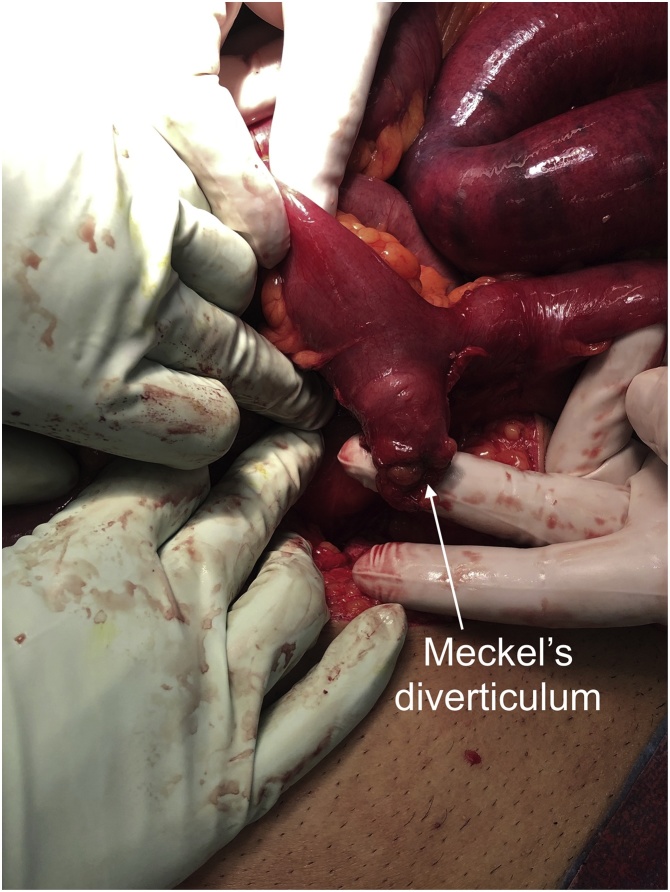
Photograph of the laparotomy with the section of ileum containing a Meckel’s diverticulum.

**Fig. 7:**
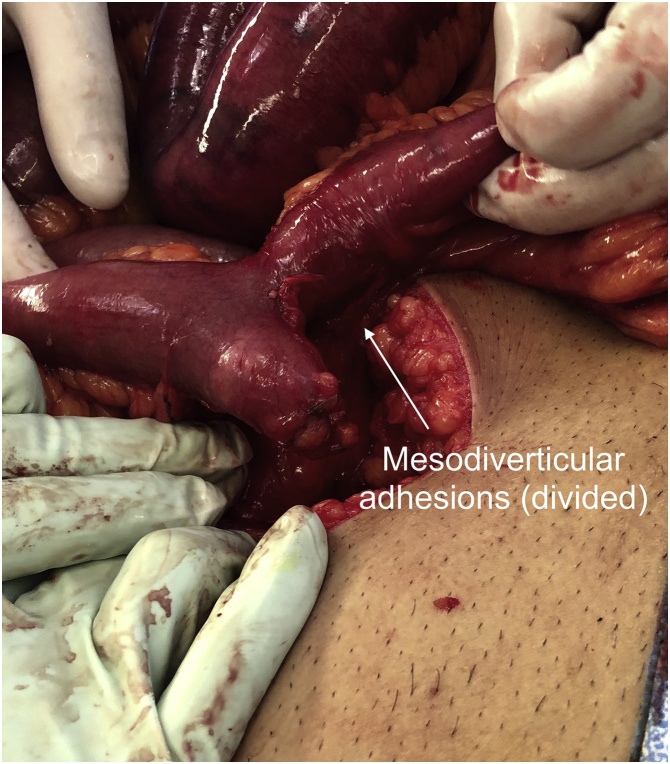
Photograph of the mesodiverticular adhesions divided from the small bowel, there is residual imprinting of the adhesions onto the proximal portion of the small bowel adjacent to the Meckel’s diverticulum. This indicates the site of the transition point.

**Fig. 8:**
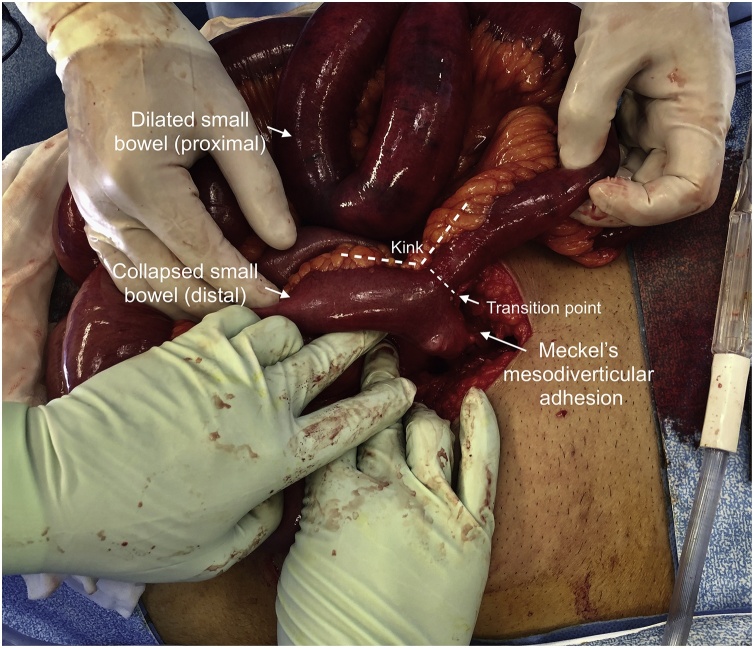
Photograph of the laparotomy with the patient viewed (left to right) from head to toe. The proximal small bowel is dilated and congested, the distal small bowel is collapsed. The transition point is angulated and marked by dotted lines. The kink is formed by the Meckel’s mesodiverticular adhesions (divided).

During the laparotomy, it was revealed he had a 4 cm Meckel’s diverticulum (Fig. 6) 20 cm proximal to the ileocaecal valve which had somehow become adhered to its own mesentery; this mesodiverticular adhesion (Fig. 7) had caused a kink in the lumen and obstruction of the proximal limb of the small bowel (Fig. 8). The extensively scarred section of Meckel’s diverticulum along with the adjacent small bowel segment was resected and a side-to-side hand-sewn anastomosis was fashioned. The specimen was sent for histopathology which confirmed the diagnosis of a true Meckel’s diverticulum of the small bowel containing typical villous mucosa with a small focal area of ulceration and associated prominent follicular lymphoid hyperplasia. The patient made an uneventful recovery, was discharged home day 3 postoperatively, and had no issues on follow up in the outpatient clinic.

## 3 Discussion

Meckel’s diverticulum was originally described by Fabricius Hildanus in 1598. However, it is named after Johann Friedrich Meckel, who established its embryonic origin in 1809. Meckel’s diverticulum is the most common congenital anomaly of the small intestine, with a prevalence of approximately 1–3%, It is a true diverticulum containing all layers of the bowel wall. The average length of a Meckel’s diverticulum is 3 cm but may range between 1 cm and 10 cm. Meckel’s diverticulum is usually found within 100 cm of the ileocaecal valve on the antimesenteric border of the ileum. The mean distance from the ileocaecal valve varies with age; the average distance for children under 2 years of age is 34 cm; and for adults, 67 cm. Most cases of Meckel’s diverticulum are asymptomatic, with the estimated risk of developing lifetime complications being about 4% [6].

Diagnosis of Meckel’s diverticulum is difficult to confirm preoperatively as most patients are asymptomatic. Among the symptomatic patients, two types of heterotopic mucosa (gastric and pancreatic) are found histologically within the diverticula. Frequent complications of Meckel’s diverticulum include hemorrhage, intestinal obstruction, and infection (diverticulitis). Intestinal obstruction is the second most common complication of Meckel’s diverticulum [7]. There are numerous proposed mechanisms for bowel obstruction arising from a Meckel’s diverticulum. Obstruction can be caused by trapping of a bowel loop by a mesodiverticular band; volvulus of the diverticulum around a mesodiverticular band; intussusception, as well as by an extension into a hernia sac (Littre’s hernia) [1,2].

Various imaging modalities have been used for diagnosing Meckel’s diverticulum. Conventional radiographic examination is of limited value. Ultrasonography has some use in the investigation of Meckel’s diverticulum; with high-resolution sonography able to demonstrate a fluid-filled structure in the right lower quadrant having the appearance of a blind-ending thick-walled loop of bowel [8]. Computed tomography (CT) has limited use in identifying a Meckel’s diverticulum as it is difficult to distinguish from normal small bowel in uncomplicated cases. However, visualisation of a blind-ending fluid or gas-filled structure in continuity with the small bowel on CT may suggest the presence of a Meckel’s diverticulum [9].

It remains controversial whether all incidental Meckel’s diverticula should be resected in asymptomatic individuals [10]. On the other hand, treatment for symptomatic patients should always include resection of the diverticulum or the segment of the bowel affected by the pathology [11].

## 4 Conclusion

Although Meckel’s diverticulum is the most prevalent congenital abnormality of the gastrointestinal tract; it is often difficult to diagnose due to the absence of symptoms in most patients. The complications of Meckel’s diverticulum should be considered by the treating clinician in the differential diagnosis of small bowel obstruction.

## Declaration of Competing Interest

There are no conflicts of interest.

## Funding

There are no sources of funding for this case report.

## Ethical approval

This case report is not subject to ethics approval at our institution.

## Consent

Verbal and written consent has been obtained from the patient who has also been de-identified.

## Authors contribution

Lei Ying – case report concept and writing, photographs and figures.

John Yahng – literature review.

## Registration of research studies

NA.

## Guarantor

Lei Ying.

## Provenance and peer review

Not commissioned, externally peer-reviewed.

## References

[1] Sagar J., Kumar V., Shah D.K. (2006). Meckel’s diverticulum: a systematic review. J. R. Soc. Med..

[2] Yahchouchy E.K., Marano A.F., Etienne J.C., Fingerhut A.L. (2001). Meckel’s diverticulum. J. Am. Coll. Surg..

[3] Francis A., Kantarovich D., Khoshnam N., Alazraki A.L., Patel B., Shehata B.M. (2016). Pediatric Meckel’s diverticulum: report of 208 cases and review of the literature. Fetal Pediatr. Pathol..

[4] Bani-Hani K.E., Shatnawi N.J. (2004). Meckel’s diverticulum: comparison of incidental and symptomatic cases. World J. Surg..

[5] Agha R.A., Fowler A.J., Saetta A., Barai I., Rajmohan S., Orgill D.P., for the SCARE Group (2018). The SCARE statement: consensus-based surgical case report guidelines. Int. J. Surg..

[6] Whang E.E., Ashley S.W., Zimmer M.J., Brunicardi F. Charles (2005). Small intestine. Schwart’s Principles of Surgery.

[7] Leijonmarck C.E., Bonman-Sandelin K., Frisell J., Räf L. (1986). Meckel’s diverticulum in the adult. Br. J. Surg..

[8] Elsayes K.M., Menias C.O., Harvin H.J., Francis I.R. (2007). Imaging manifestations of Meckel’s diverticulum. AJR Am. J. Roentgenol..

[9] Won Y., Lee H.W., Ku Y.M., Lee S.L., Seo K.J., Lee J.I. (2016). Multidetector-row computed tomography (MDCT) features of small bowel obstruction (SBO) caused by Meckel’s diverticulum. Diagn. Interv. Imaging.

[10] Weinstein E.C., Cain J.C., Remine W.H. (1962). Meckel’s diverticulum: 55 years of clinical and surgical experience. JAMA.

[11] Varcoe R.L., Wong S.W., Taylor C.F., Newstead G.L. (2004). Diverticulectomy is inadequate treatment for short Meckel’s diverticulum with heterotopic mucosa. ANZ J. Surg..

